# Serum level and immunohistochemical expression of vascular endothelial growth factor for the prediction of postoperative recurrence in renal cell carcinoma

**DOI:** 10.1186/1756-0500-7-369

**Published:** 2014-06-17

**Authors:** Naoyuki Fujita, Takatsugu Okegawa, Yuichi Terado, Mitsuhiro Tambo, Eiji Higashihara, Kikuo Nutahara

**Affiliations:** 1 Departments of Urology, Kyorin University School of Medicine, 6-20-2 Shinkawa, Mitaka, Tokyo, Japan; 2 Departments of Pathology, Kyorin University School of Medicine, 6-20-2 Shinkawa, Mitaka, Tokyo, Japan

**Keywords:** Recurrence, Renal cell carcinoma, Vascular endothelial growth factor, Nephrectomy

## Abstract

**Background:**

Vascular endothelial growth factor (VEGF) plays a major role in angiogenesis. One of the functions of VEGF is to regulate neovascularization in clear cell renal cell carcinoma (CCRCC). The objective of our study was to examine whether before nephrectomy serum levels of VEGF or expression of VEGF using immunohistochemistry (IHC) could predict postoperative recurrence in nonmetastatic CCRCC.

**Results:**

Twelve patients (14.5%) had recurrence during a mean follow-up of 52.6 ± 31.2 months. The serum VEGF level was significantly higher in patients with recurrence than in those without recurrence (*P* = 0.038). High serum VEGF levels were above 416 pg/mL; this value was chosen based on a receiver operating characteristic analysis. The recurrence-free survival rate in patients with a high serum VEGF level was significantly lower than in those with a low serum VEGF level (*P* = 0.003). In total, tumors from 26 patients (31.3%) showed overexpression of VEGF using IHC. The recurrence-free survival rate in the IHC-positive group was significantly lower than that in the IHC-negative group (*P* = 0.044). Multivariate analysis indicated that preoperative serum VEGF levels (*P* = 0.013) and female gender (*P* = 0.004) were independent predictors of postoperative recurrence in nonmetastatic CCRCC.

**Conclusions:**

Preoperative serum VEGF levels is a useful predictor compared with IHC analysis of VEGF of postoperative recurrence in nonmetastatic CCRCC.

## Background

Clear cell renal cell carcinoma (CCRCC) is characterized by inactivation of the von Hipple-Lindau (VHL) pathway caused by somatic mutations or methylation of the VHL gene in the majority of patients. The VHL gene product is involved in the regulation of a transcription factor called hypoxia-inducible factor (HIF), which is a heterodimer of an α and β subunit. In cells with deficient or aberrant VHL protein, HIFα accumulates and binds to HIFβ. The resultant increased production of vascular endothelial growth factor (VEGF) is considered to be fundamental to the highly angiogenic nature of CCRCC and critical to oncogenesis [1,2]. Anti-angiogenesis treatment for CCRCC includes the use of several inhibitors of VEGF and its cognate receptor VEGF receptor (VEGFR) 2 that have dramatic antitumor activity in CCRCC [3]. Several studies have reported that some molecular markers (tissue Ki-67, tissue p53, tissue VEGFR-1, tissue VEGF-D and serum carbonic anhydrase IX) could predict disease-free survival after nephrectomy for nonmetastatic CCRCC [4,5].

Studies have shown that serum levels of VEGF and tumor VEGF expression are useful predictors of prognosis in RCC [6-8]. However, to our knowledge, there have been very few reports regarding the association between VEGF and recurrence after nephrectomy in nonmetastatic CCRCC [5,9]. Previously, we reported that the pretreatment serum level of VEGF correlated with postoperative recurrence in patients with nonmetastatic CCRCC [9]. In the present study, we evaluated whether preoperative serum VEGF levels and expression of VEGF, assessed using immunohistochemistry (IHC), could predict postoperative recurrence in patients with nonmetastatic CCRCC. The discovery of reliable biomarkers in CCRCC could have an important impact on diagnosis, prognosis and prediction of therapeutic benefit.

## Methods

### Patients

A total of 83 patients with nonmetastatic CCRCC, who underwent radical nephrectomy at the Kyorin University Hospital between August 2001 and March 2010, were enrolled in the study. Initial clinical stage determination consisted of a physical examination, computed tomography (CT) and a bone scan. Data on variables such as age, gender, tumor stage, pathological grade, histological vein invasion, tumor size, tumor necrosis, Eastern Cooperative Oncology Group performance status (ECOG PS), symptoms (flank pain, flank mass, and hematuria), UCLA Integrated Staging System (UISS) [10] and biochemical parameters [e.g., levels of hemoglobin (Hb), lactate dehydrogenase (LDH), calcium (Ca) and C-reactive protein (CRP)] were collected. Tumor stage was determined on the basis of the 2009 TNM classification of the Union for International Cancer Control. Pathological grade was determined on the basis of the General Rules for Clinical and Pathological Studies on Renal Cell Carcinoma in Japan (pathological grade was classified as G1–G3). UISS incorporates the 1997 TNM classification, ECOG PS and pathological grade [10]. The cut-off level of LDH was determined to be 1.5 times the upper limit of normal (ULN). Corrected serum Ca was calculated as follows: total Ca + (4 − albumin). Tumor size, CRP and anemia were dichotomized as follows: tumor size, <5 cm vs. ≥5 cm, CRP, ≤1.0 vs. >1.0 mg/dL, and anemia, ≤13 vs. >13 g/dL in males and ≤11.5 vs. >11.5 g/dL in females [11,12]. The Ethics Committee of the University of Kyorin approved this study. Informed consent was obtained from all patients.

### Blood samples

Serum samples were obtained before nephrectomy from a peripheral vein. An 8.5 mL blood sample was taken and after clotting, the blood sample was centrifuged at 2000 rpm for 10 min. Serum was then stored at -80°C in 1 mL aliquots until analysis. A VEGF assay was performed using a commercial quantitative immunoassay kit for human VEGF165 (Quantikine, Human VEGF immunoassay, R&S Systems, Minneapolis, MN, USA). Serum samples (100 μL) were added to a 96-well microtiter plate coated with purified antihuman VEGF mouse IgG monoclonal antibodies and incubated at room temperature for 2 h. After washing, 200 μL of the secondary antibody solution, containing VEGF-specific polyclonal goat antibodies, was added and incubated for 2 h at room temperature. Substrate solution was added and the reaction continued for 25 min. Absorbance was measured at 450 nm using a microtiter plate reader (EMax, Molecular Devices, Sunnyvale, CA, USA). The sensitivity of the assay was 9 pg/ml, and coefficients of variance ranged from 4.5–6.7% and from 6.2–8.8% within and between assays, respectively.

### Tissue samples

Paraffin block sections were available for all cases. Representative hematoxylin and eosin-stained specimens were reviewed to assess the histological type, histological vein invasion, tumor necrosis and pathological grade as well as to select sections for IHC. Cancer tissue samples for IHC analysis were selected from the site of highest pathological grade.

### IHC

IHC was performed on 5 μm paraffin-embedded tissue sections placed on poly-l-lysine-coated glass slides. After heat drying, sections were deparaffinized in xylene and rehydrated in a series of graded ethanol solutions. Endogenous peroxidase was blocked with 0.3% hydrogen peroxide for 5 min at room temperature. For antigen retrieval, sections were immersed in a 0.01 mol/L sodium citrate buffer (pH 6.0), heated in a 600 W microwave for 4 min (twice) and allowed to cool for 30 min at room temperature. The slides were incubated at room temperature for 30 min with a polyclonal anti-VEGF antibody that detects the 189-, 165-, and 121-amino acid splice variants of human VEGF (sc-152; titer, 1:100; Santa Cruz Biotechnology, Santa Cruz, CA, USA). A biotin-streptavidin detection system (Dako, Glostrup, Denmark) was subsequently applied using diaminobenzidine as the chromogen. As a negative control, phosphate-buffered saline or a normal rabbit immunoglobulin fraction diluted to the same protein concentration as the primary antibody was used. A pathologist independently evaluated the immunostaining. Staining was semiquantitatively assessed on the basis of a four-grade scale (0, absence of faint membrane staining in most tumor cells (<20%); 1+, membrane staining of most tumor cells; 2+, diffuse membrane staining and cytoplasmic staining of groups of tumor cells (<50%); and 3+, significant cytoplasmic staining in most cells). A score of 2+ or above was considered positive [7].

### Statistics

Variables were compared between the different groups using the Mann-Whitney *U* and Kruskal-Wallis tests. Independence of fitness of the categorical data was estimated using the χ^2^ test. Recurrence-free survival rates were calculated from the date of nephrectomy to the recognized date of recurrence. The recurrence-free survival rate was calculated using the Kaplan-Meier method, and the significance of comparisons between groups was measured using the log-rank test. A multivariate analysis was performed using a Cox proportional hazards regression model. The optimal serum VEGF value to discriminate recurrence, calculated using receiver operating characteristic (ROC) curve analysis. *P*-values of <0.05 were considered statistically significant. All statistical analyses were performed using statistical software available commercially (SPSS v.18.0).

## Results

Patient characteristics are shown in Table 1. Mean age of the patients was 65.0 ± 12.4 years. The mean follow-up duration was 52.6 ± 31.2 months (range, 3–139 months). Twelve patients (14.5%) had recurrence after surgery. The recurrence sites were as follows: lung (9 patients, 75%), bone (2 patients, 17%) and retroperitoneum (1 patient, 8%). In the recurrence group, four patients (33.3%) were treated with molecular-targeted therapy and two patients (16.7%) were treated with interferon therapy after recurrence. Two patients were performed prophylactic interferon therapy for one year, and they did not have recurrence. There was no significant association between prophylactic interferon therapy and recurrence (*P* = 0.556). Tumor stage, pathological grade, tumor necrosis, UISS, gender, hemoglobin, serum VEGF level and VEGF IHC-expression were significantly associated with recurrence (Table 1).

**Table 1:** Patient characteristics in relation to without and with recurrence CCRCC

**Characteristic**	**Total**	**Without recurrence group**	**With recurrence group**	**P-value**
	**(n = 83)**	**(n = 71)**	**(n = 12)**	
Age (years)				
Median [range]	67 [23-83]	67 [23-83]	65 [44-82]	0.820
Gender (%)				
Male	52 (63)	48 (68)	4 (33)	
Female	31 (37)	23 (32)	8 (67)	0.049
ECOG PS (%)				
0	81 (98)	70 (99)	11 (92)	
1	1 (1)	1 (1)	0 (0)	
2	1 (1)	0 (0)	1 (8)	0.046
T stage (%)				
T1	64 (77)	60 (85)	4 (33)	
T2	5 (6)	4 (5)	1 (9)	
T3	14 (17)	7 (10)	7 (58)	
T4	0 (0)	0 (0)	0 (0)	<0.001
Grade (%)				
1	7 (8)	5 (7)	2 (17)	
2	70 (84)	64 (90)	6 (50)	
3	6 (8)	2 (3)	4 (33)	<0.001
UISS risk classification (%)				
Low risk	61 (73)	58 (82)	3 (25)	
Intermediate risk	21 (25)	13 (18)	8 (67)	
High risk	1 (2)	0 (0)	1 (8)	<0.001
Histological vein invasion (%)				
No	53 (64)	48 (68)	5 (42)	
Yes	30 (36)	23 (32)	7 (58)	0.083
Tumor necrosis (%)				
No	70 (84)	64 (90)	6 (50)	
Yes	13 (16)	7 (10)	6 (50)	<0.001
Symptoms (%)				
No	59 (71)	52 (73)	7 (58)	
Yes	24 (29)	19 (27)	5 (42)	0.292
IHC staining (%)				
Grade 0	39 (47)	37 (52)	2 (17)	
Grade 1+	18 (22)	15 (21)	3 (25)	
Grade 2+	19 (23)	15 (21)	4 (33)	
Grade 3+	7 (8)	4 (6)	3 (25)	0.045
Tumor size (mm)				
Median [range]	40.0 [12-120]	40.0 [12-90]	43.5 [30-120]	0.118
Hemoglobin (g/dL )				
Median [range]	13.4 [8.1-17.0]	13.55 [9.6-17.0]	12.2 [8.1-14.7]	0.014
Corrected Ca (mg/dL)				
Median [range]	9.30 [8.5-11.2]	9.25 [8.5-10.8]	9.35 [8.6-11.2]	0.606
CRP (mg/dL)				
Median [range]	0.20 [0.0-12.4]	0.20 [0.0-12.4]	0.35 [0.0-6.2]	0.335
Serum VEGF (pg/mL)				
Median [range]	299 [42-1460]	290 [42-1460]	478 [140-982]	0.038

**Figure 1:**
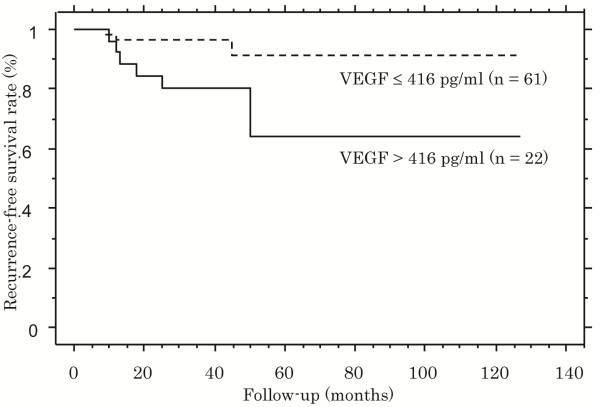
**Kaplan-Meier analysis of recurrence-free survival rates: comparison between patients with high and low serum VEGF levels.** VEGF concentrations were determined before surgery. The solid line represents serum VEGF >416 pg/mL (n = 22) and the dotted line represents serum VEGF ≤416 pg/mL (n = 61). Patients with VEGF >416 pg/mL had a significantly increased risk of recurrence compared with those with VEGF ≤416 pg/mL (*P* = 0.003).

The median serum level of VEGF in 83 patients was 299 pg/mL (range, 42–1460 pg/mL). The serum VEGF level in patients with recurrence was significantly higher than in those without recurrence (*P* = 0.038). The serum VEGF level was significantly associated with pathological grade (*P* = 0.024), however, it was not significantly associated with tumor stage, histological vein invasion or UISS risk classification (*P* = 0.109, 0.193, and 0.282, respectively) (Table 2). The cut-off value (416 pg/mL) of serum VEGF was determined using ROC analysis. Patients with a high serum VEGF level had significantly lower recurrence-free survival rates than those with a low serum VEGF level (*P* = 0.003; Figure 1).

**Table 2:** Patient characteristics in relation to serum VEGF levels and IHC tissue staining of VEGF

**Characteristic**	**VEGF levels**		**IHC tissue staining of VEGF**		
	**(pg/mL)**		**Positive group**	**Negative group**	
	**Median [range]**	**P-value**	**n (%)**	**n (%)**	**P-value**
Patient			26	57	
Gender					
Male	314[42-1460]		13 (50)	39 (68)	
Female	290[52-912]	0.890	13 (50)	18 (32)	0.107
T stage					
T1	313[42-1330]		16 (65)	48 (84)	
T2	140[50-353]		4 (15)	1 (2)	
T3	294[79-1460]		6 (23)	8 (14)	
T4	-	0.109	0 (0)	0 (0)	0.023
Grade					
1	519[290-912]		1 (4)	6 (11)	
2	283[42-1460]		19 (73)	51 (89)	
3	385[225-982]	0.024	6 (23)	0 (0)	<0.001
UISS risk classification					
Low risk	313[42-1330]		14 (57)	47 (82)	
Intermediate risk	282[50-1460]		11 (42)	10 (18)	
High risk	982	0.282	1 (4)	0 (0)	0.014
Histological vein invasion					
No	325[42-1460]		15 (58)	38 (67)	
Yes	279[79-1330]	0.198	11 (42)	19 (33)	0.430
Tumor necrosis					
No	288[42-1460]		22 (85)	48 (84)	
Yes	405[93-982]	0.153	4 (15)	9 (16)	0.962
IHC staining					
Grade 0	313 [42-968]		-	-	
Grade 1+	325 [91-511]		-	-	
Grade 2+	248 [50-1460]		-	-	
Grade 3+	507 [140-1330]	0.378	-	-	
Recurrence					
No	290[42-1460]		19 (73)	52 (91)	
Yes	478[140-982]	0.038	7 (27)	5 (9)	0.043

**Figure 2:**
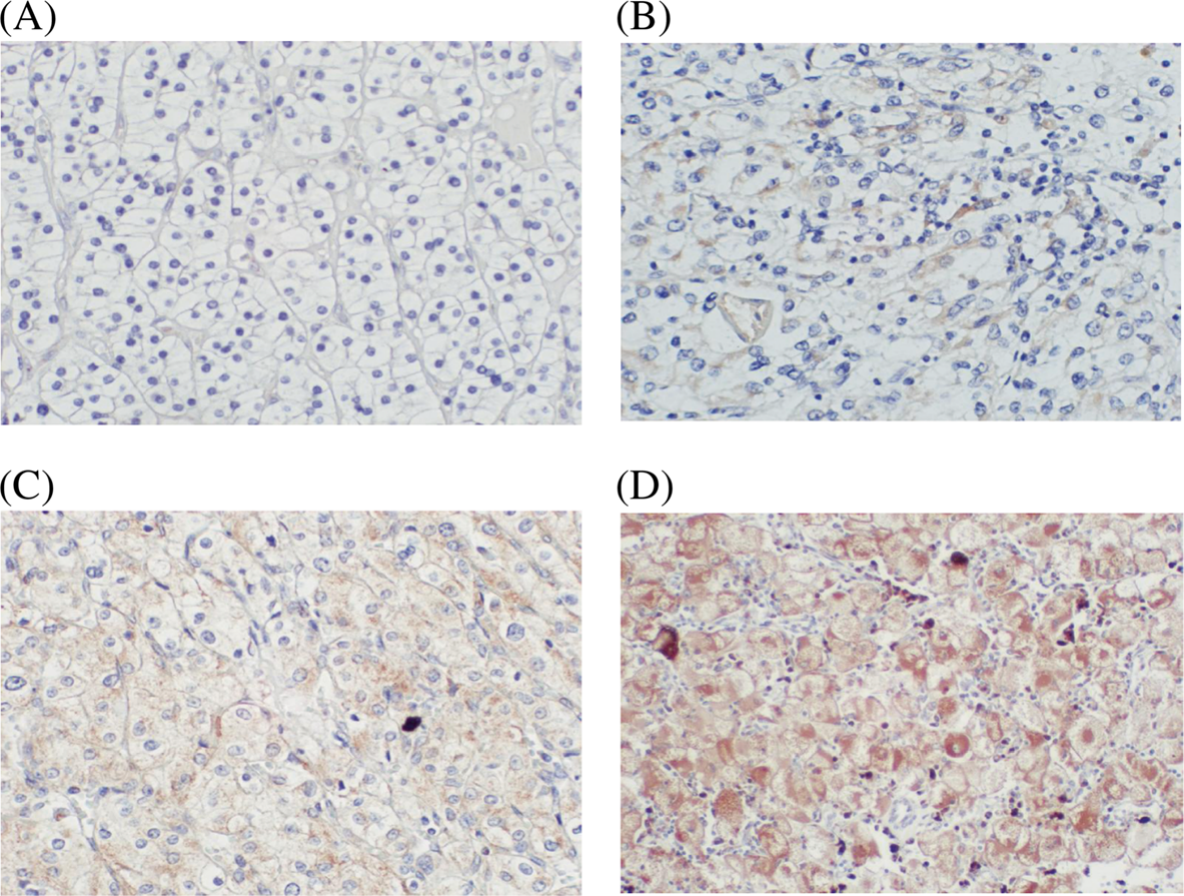
**IHC staining of VEGF in CCRCC. (A)** No staining or membrane staining was observed in <20% of the tumor cells (grade 0); **(B)** Membrane without cytoplasmic immunostaining of tumor cells (grade 1+); **(C)** Diffuse membrane and cytoplasmic staining in <50% of tumor cells (grade 2+); and **(D)** Significant cytoplasmic staining in most tumor cells (grade 3+). Magnification was 400 × .

**Figure 3:**
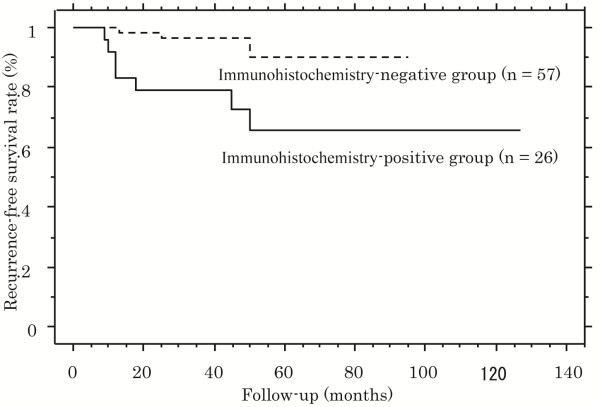
**Kaplan-Meier analysis of recurrence-free survival rates: comparison between patients positive and negative for VEGF expression using IHC.** The solid line represents the IHC-positive group (n = 26), and the dotted line represents the IHC-negative group (n = 57). The IHC-positive group had a significantly increased risk of recurrence compared with the IHC-negative group (*P* = 0.044)

Expression of VEGF using IHC is shown in Figure 2. Twenty-six patients (31.3%) were positive for VEGF expression. There was no significant association between serum levels and IHC-expression of VEGF (IHC expression Grade 0: 313 pg/mL, Grade 1+: 325 pg/mL, Grade 2+: 248 pg/mL, Grade 3+: 507 pg/mL; *P* = 0.378). VEGF IHC-expression was significantly associated with tumor stage, tumor grade, UISS risk classification and recurrence (Table 2). Patients positive for VEGF had significantly lower recurrence-free survival rates than those negative for VEGF IHC-expression (*P* = 0.044; Figure 3).

Tumor stage, pathological grade, histological vein invasion, tumor necrosis, UISS, female gender, anemia, CRP, serum VEGF level and VEGF IHC-expression were significant univariate factors in predicting postoperative recurrence. A multivariate Cox logistic regression analysis showed that the preoperative serum level of VEGF and female gender were the only independent predictors of recurrence after radical nephrectomy (Table 3).

**Table 3:** Univariate and multivariate analyses to predict postoperative recurrence

**Variable**		**Univariate**			**Multivariate**	
		**Recurrence**				
		**n**	**n (%)**	***P*-value**	**Hazard ratio (95% CI)**	***P*-value**
T stage	1 + 2	69	5 (7)		1.0 (ref)	
	3 + 4	14	7 (50)	<0.001	4.376 (0.186–103.189)	0.360
Grade	1 + 2	77	8 (10)		1.0 (ref)	
	3	6	4 (67)	<0.001	2.224 (0.091–54.113)	0.624
Histological	No	53	5 (9)		1.0 (ref)	
Vein invasion	Yes	30	7 (23)	0.033	1.585 (0.248–10.120)	0.626
Tumor size	<5 cm	54	7 (13)			
	≥5 cm	29	5 (17)	0.549		
Symptoms	No	59	7 (12)			
	Yes	24	5 (21)	0.195		
Tumor necrosis	No	70	6 (9)		1.0 (ref)	
	Yes	13	6 (46)	<0.001	4.023 (0.523–30.944)	0.181
UISS	Low	61	3 (5)		1.0 (ref)	
	Intermediate + high	22	9 (41)	<0.001	7.373 (0.434–125.320)	0.170
Gender	Male	52	4 (8)		1.0 (ref)	
	Female	31	8 (26)	0.017	13.869 (2.354–81.713)	0.004
ECOG PS	0	81	11 (14)			
	≥1	2	1 (50)	0.146		
Age	<65	48	6 (12)			
(years)	≥65	35	6 (17)	0.809		
Anemia	No	60	6 (10)		1.0 (ref)	
	Yes	22	6 (27)	0.035	1.536 (0.197–11.972)	0.682
	Missing	1				
LDH	≤1.5 × ULN	71	11 (15)			
	>1.5 × ULN	2	0 (0)	NA		
	Missing	10				
Corrected Ca	≤10	71	10 (14)			
(mg/dL)	>10	8	1 (12)	0.781		
	Missing	4				
CRP	≤1.0	70	8 (11)		1.0 (ref)	
(mg/dL)	>1.0	12	4 (33)	0.015	0.476 (0.032–7.168)	0.591
	Missing	1				
Serum VEGF	≤416	61	4 (7)		1.0 (ref)	
(pg/mL)	>416	22	8 (36)	0.003	18.059 (1.853–176.019)	0.013
IHC staining	Negative	57	5 (9)		1.0 (ref)	
	Positive	26	7 (27)	0.044	1.130 (0.160–7.967)	0.902

NA: not available, ref: reference.

## Discussion

Angiogenesis is essential for tumor development and metastasis. VEGF plays a major role in stimulating the formation of new blood vessels in several biological processes including tumor angiogenesis [13]. CCRCC is a highly angiogenic tumor that secretes VEGF [14]. The Kattan postoperative nomogram and UISS are widely used to predict tumor recurrence after treatment of RCC [10,15]; however, improved methods of prediction are required, and attempts to find better prognostic criteria are ongoing. This study showed that preoperative serum levels of VEGF may be a useful predictor of postoperative recurrence in patients with nonmetastatic CCRCC.

Rioux-Leclercq *et al.* reported that the expression of VEGF, assessed using IHC, in CCRCC tumors from 50 patients with a median follow-up of 11 months, was significantly associated with plasma VEGF levels, measured using an enzyme-linked immunoassay. Both expression of VEGF, using IHC, and plasma VEGF levels were significantly correlated with Fuhrman grade and tumor stage. VEGF IHC-expression correlated significantly with progression (*P* < 0.01); however, there was insignificant difference in plasma VEGF levels between patients with and without progression (*P* = 0.06) [16].

There are several possible explanations for the discrepancy between the study by Rioux-Leclercq *et al.* and our study. First, both the studies were evaluated using a small sample size and short follow-up period. Second, tumor size affects the amount of circulating tumor-derived VEGF [17]; therefore, serum levels of VEGF may be higher in patients with larger tumors even if tumor cells express a similar level of VEGF. Third, variability in VEGF isoforms may affect the correlation between serum VEGF levels and expression of VEGF using IHC. VEGF has five isoforms (VEGF206, 189, 165, 145 and 121). In the present study, only VEGF189, 165 and 121 were assessed using IHC because high expression of these forms has been reported in RCC [18]. VEGF165 was selected for serum determination because VEGF165 and VEGF121 are the predominant isoforms secreted by most tumors [19]. The relationship between the pattern of VEGF isoforms synthesized in tumors and their concentration in the circulation remains unclear and warrants further study. Fourthly, bias could exist in assessing VEGF levels in plasma or serum samples. Serum samples contain high levels of VEGF due to its release by activated platelets during clotting [20]. Several studies reported a correlation between platelet counts and serum VEGF, and higher serum VEGF levels per platelet in cancer patients [21,22]. Niers *et al.* reported that elevated plasma VEGF levels in blood samples were highly dependent on the method of collection and platelet VEGF content. Therefore, for the purpose of avoiding platelet activation *ex vivo*, blood samples were collected drop by drop without using a tourniquet [23].

Studies have shown that elevated serum levels of VEGF are associated with disease prognosis [6,24]. Previously, we reported that the pretreatment serum level of VEGF correlated with postoperative recurrence in patients with nonmetastatic CCRCC [9]. In this study, we extended the mean period of follow-up (33.9 ± 27.2 to 52.6 ± 31.2 months), and evaluated VEGF using both serum analysis and immunohistochemical assay. The number of patients with recurrence increased from 8 to 12, but the cut-off value using ROC analysis was the same. Jacobsen *et al.* reported that serum levels of VEGF, assessed using a cut-off value of 343.5 pg/mL, as determined using the median value measured in 164 patients with RCC including various histological subtypes, significantly correlated with tumor stage, pathological grade and prognosis [6]. On the other hand, Tanimoto *et al.* reported that serum VEGF levels measured using the same methodology as in the Jacobsen *et al.* study and assessed using a cut-off value (400 pg/mL), as determined using ROC analysis in 45 patients with CCRCC, were not significantly correlated with tumor stage, pathological grade, tumor size or prognosis [25]. With regard to the relationship between histological subtype and serum levels of VEGF, there was no significant difference in serum VEGF levels between papillary RCC and CCRCC. However, serum VEGF levels in chromophobe RCC were found to be significantly lower than those in CCRCC [6]. The discordant result may be attributed to differences in RCC histological subtypes in subjects and the method used to calculate the cut-off value.

Based on IHC data, several investigators have reported that VEGF overexpression in CCRCC was associated with tumor stage, pathological grade, histological vein invasion and prognosis [7,8]. In contrast, Verheul *et al.* reported that VEGF expression using IHC in CCRCC was not significantly correlated with prognosis [26]. This discrepancy in IHC results could be due to several factors including differences in fixation, scoring and staining methods [7,8,26].

Predictive factors of recurrence after nephrectomy in patients with RCC include anatomical (TNM classification), histological (pathological grade, histological vein invasion and tumor necrosis), clinical (symptoms and performance status) and biochemical (level of CRP) features [10-12,15,27,28]. A major prognostic model for localized RCC is the UISS that combines TNM stage, Fuhrman grade and ECOG PS [10]. In our study, symptoms, ECOG PS and tumor size were not significant predictive factors of postoperative recurrence in patients with nonmetastatic CCRCC. The present study showed that female patients had a significantly poorer recurrence-free survival rate than male patients. However, Leibovich *et al.* reported no significant relationship between gender and prognosis in 1671 patients with CCRCC [27]. The gender difference in postoperative recurrence in our study may be due to the small number of enrolled patients and the retrospective nature of our study.

In RCC, few studies have investigated VEGF as a predictor of postoperative recurrence. However, several studies in patients with esophageal, hepatocellular and colorectal carcinomas showed that elevation of serum VEGF levels might be a predictor for poor recurrence-free survival [29-31]. The present study had some limitations. First, the relatively small sample size may produce false-positive results, or over-estimate the magnitude of an association. Second, the short follow-up time may be insufficient to observe recurrence. Third, this was a retrospective study, which was susceptible to selection bias. A larger study and one with more balanced groups (i.e. patients with and without recurrence) will produce more precise estimates. Further large prospective studies are needed to confirm our results.

## Conclusions

Our study showed that measurement of preoperative VEGF serum levels in patients with nonmetastatic CCRCC may be more useful than using IHC to assess VEGF expression in tumor tissues for prediction of postoperative recurrence after nephrectomy.

## Abbreviations

Ca: Calcium; CCRCC: Clear cell renal cell carcinoma; CI: Confidence interval; CRP: C-reactive protein; CT: Computed tomography; ECOG PS: Eastern Cooperative Oncology Group performance status; Hb: Hemoglobin; HIF: Hypoxia-inducible factor; IHC: Immunohistochemistry; LDH: Lactate dehydrogenase; RCC: Renal cell carcinoma; ROC: Receiver operating characteristic; SD: Standard deviation; UISS: UCLA Integrated Staging System; ULN: Upper limit of normal; VEGF: Vascular endothelial growth factor; VEGFR: Vascular endothelial growth factor receptor.

## Competing interests

The authors declare that they have no competing interests.

## Authors’ contributions

NF and TO designed the research. NF, TO and YT obtained the data. YT provided the pathological data. NF, TO, YT and MT analyzed the data. NF and MT wrote the manuscript. TO, EH and KN have made contributions providing critical review of the manuscript for important intellectual content. All authors read and approved the final manuscript.
